# Does maternal health voucher scheme have association with distance inequality in maternal and newborn care utilization? Evidence from rural Bangladesh

**DOI:** 10.1371/journal.pone.0295306

**Published:** 2023-12-07

**Authors:** Asiful Haidar Chowdhury, Syed Manzoor Ahmed Hanifi, Mohammad Iqbal, Aazia Hossain, William Stones, Mark Amos, Saseendran Pallikadavath, Abbas Bhuiya, Shehrin Shaila Mahmood

**Affiliations:** 1 International Centre for Diarrhoeal Disease Research, Dhaka, Bangladesh; 2 Health System and Population Studies Division, Dhaka, Bangladesh; 3 Kamuzu University of Health Sciences, Blantyre, Malawi; 4 Portsmouth Brawajaya Centre for Global Health, Population and Policy, University of Portsmouth, Portsmouth, United Kingdom; Imperial College London School of Public Health, EGYPT

## Abstract

**Background:**

Bangladesh has made substantial progress in maternal health. However, persistent inequities in service use undermine the achievements at the national level. In 2007, the government introduced a Maternal Health Voucher Scheme (MHVS) to reduce barriers to service utilization. The current study explores the impact of MHVS on reducing distance inequality in utilization across the maternal and newborn continuum of care (MNCoC).

**Methods:**

A cross-sectional survey was conducted from October’2017 to April’2018 in four selected MHVS sub-districts of Chattogram and Sylhet Divisions of Bangladesh. 2,400 women with at-least one child aged below two years were randomly selected. Both bivariate and multivariate analyses were carried out to explore the absolute and relative influence of the voucher scheme and chi-square test was used for hypothesis testing.

**Results:**

Nineteen percent of the women were MHVS beneficiaries and 23% of them lived within 5 km of the health facility. Among the beneficiaries no significant differences were observed in the utilization of at-least 4 antenatal visits, skilled-assistance at delivery, postnatal care, and MNCoC between those living closer to the health facility and those living far away. However, a higher facility delivery rate was observed among beneficiary women living closer. By contrast, for non-beneficiaries, a significant difference was found in service use between women living closer to health facilities compared to those living further away.

**Conclusion:**

The study found the use of MNCoC to be similar for all MHVS beneficiaries irrespective of their distance to health facilities whereas non-beneficiary women living further away had lower utilization rates. MHVS could have potentially reduced distance-related inequality for its beneficiaries. However, despite the provision of transport incentives under MHVS the reduction in inequality in facility delivery was limited. We propose a revision of the transportation incentive adjusting for distance, geographical remoteness, road condition, and transport cost to enhance the impact of MHVS.

## Background

Over the past decade, Bangladesh has made substantial improvements in maternal and child health. The maternal mortality ratio in the country has declined from 574 in 1990 [1] to 196 per 100,000 live births in 2016 [2]. The neonatal mortality rate decreased from 56.4 per 1000 live births in 1990 to 30 per 1000 live births in 2015 [3]. Despite the progress, considerable challenges remain in terms of ensuring comprehensive access to maternal and neonatal health services for all. The National Demographic and Health Survey of Bangladesh (BDHS) 2022 reveals that although in-facility delivery increased from 37% in 2014 to 65% in 2022, home delivery by informal healthcare providers (e.g. traditional birth attendants) still remains a significant choice of care for the majority of women [4]. This poses an additional risk to pregnant women as home-based skilled care is not supported by the existing health system structure [5]. This gap in access to skilled care disproportionately affects different population sub-groups, such as the poor, less educated, and those residing far from the health facilities [5–7]. Further to this there exists a stark difference in access to maternal care between the rural and urban populations of the country. BDHS 2022 [4] showed that women who live in urban areas are more likely to receive four or more antenatal care (ANC) (56.9% vs. 34.5%) and deliver at health facilities (76.3% vs. 60.5%) compared to their urban counterpart. The proportions of deliveries at private facilities is higher in both rural and urban areas compared to the public facilities (17.9% at public facilities and 45.9% at private facilities) [4]. Among the varied factors impacting access to quality care, distance, in particular, remains a major barrier to care-seeking in Bangladesh and it limits access to the continuum of care at all stages [8–10]. Distance operates as a barrier through two major routes [9, 11]: physical access for rural women and the costs associated with obtaining transport (e.g. van or rickshaw) to maternal care facilities. Transport costs have been identified as a major demand-side barrier to care utilization in general.

Evidence from low and middle-income countries shows that although many women visit health facilities during pregnancy for screening of risk factors, pre-existing medical conditions, and current health status, very few do this, at the intervals recommended by the World Health Organization (WHO) [12]. In Bangladesh, a national-level household survey showed about two-thirds of women (64 percent) received at least one antenatal check by a trained provider whereas only thirty-one percent of women with a live birth in the three years before the survey attended four or more antenatal visits during their last pregnancy [6]. This contributes to the persistent burden of adverse maternal and newborn outcomes. Maximum utilization of maternal, neonatal, and child health care can be anticipated to expedite progress towards the sustainable development goals (SDGs) that include multiple targets around maternal, newborn and child health [13]. Evidence from recent years suggests that access to a continuum of care (CoC) during pregnancy, childbirth, and the postnatal period has the potential to yield multiple returns on investments by reducing maternal and neonatal death as well as improving child health outcomes [14, 15].

In many developing countries programmatic interventions like voucher schemes are being tested to boost service utilization across the whole continuum of maternal and newborn care by providing cash or in-kind incentives to users as well as to providers. The schemes aim to increase service access as well as demand for services among targeted groups and are used to reduce out-of-pocket expenditure for health care for households at risk of not seeking care in the absence of the subsidy [16]. Studies have also shown that beneficiaries of the voucher scheme realize the advantage of modern medical facilities and therefore tend to seek care from qualified providers in the future as well, rather than going back to traditional or informal care providers [17]. This voucher-based system, or “demand side” financing, can serve as a substitute or complement to the traditional “supply side” approach to financing service delivery and includes a range of interventions that channel government or donor subsidies to service users rather than to service providers [16].

The government of Bangladesh introduced the Maternal Health Voucher Scheme (MHVS) in 2007 with the aim of improving maternal and child health outcomes in rural areas. Operating in 53 out of the 556 sub-districts of the country (in 2018), the voucher scheme is targeted to poor pregnant women for their first two children. To be eligible for the voucher, pregnant women need to be permanent residents of the area of intervention, landless (i.e. owning less than 6534 square feet of land), have a monthly average income of household less than 1.25 U.S. Dollars per day (equivalent monthly income less than BDT 3100). The benefits of MHVS cover three antenatal care visits, delivery at a health facility or at home with a qualified provider, one postnatal check, management of maternal complications including cesarean delivery where required, free medicines, cash allowances for transportation (flat rate of BDT 100per visit to health facility) and cash incentive to deliver at a health facility or at home with a qualified provider. The voucher can be used in both public hospitals (i.e. Upazila Health Complex (UHC), the major public health facility at the sub-district level) and designated private and non-government facilities. Provider facilities and individual staff also receive payments for each service provided to the scheme members [18–20]. Earlier reports have shown that implementation of the MHVS has substantially increased the utilization of antenatal care, skilled assistance at delivery, and postnatal care services [14]. There is also evidence for improved equity according to socioeconomic status in access to maternal health services, despite some administrative challenges in terms of disbursement of benefits [21–24]. However, evidence around the impact of MHVS on distance-related inequality is lacking. Notably it does not allow assessment of the adequacy of cash incentives provided through the scheme, in particular the transport allowance. Such an assessment can serve as an evidence base for modification of the benefit package to maximize access for beneficiaries. The current study thus focuses on the distance-related inequality in access to the continuum of maternal and neonatal healthcare among voucher recipient and non-recipient women. We examined whether utilization of services across the maternal and newborn continuum of care (CoC) varies with distance to health facilities and the extent to which the voucher scheme is able to reduce any existing inequality in maternal and newborn care use.

## Methods

### Ethical approval

Research and Ethical approval was taken from the Research Review Committee (RRC) and the Ethical Review Committee (ERC) of the International Centre for Diarrhoeal Disease Research, Bangladesh (icddr,b) before initiating the research study. Written informed consent was taken from all study participants before conducting the interviews. The consent process involved informing study participants about the study, their involvement as respondents, their right to withdraw or not to answer any of the questions, and also any risk or benefit that the study may entail.

Before conducting interviews with randomly selected women written informed consent was taken after she was informed about the study objectives and her involvement in the study.

### Study design and study site

Following a cross-sectional study design, a household-based survey was conducted from October 2017 to April 2018. A pre-tested questionnaire was used to collect the required data. The questionnaire was pretested among some MHVS and non-MHVS women in one of the study sites. Based on learning from pretesting the questionnaire wording of some of the questions was made easier to understand for the target group people. Interviewers were trained to make the question understandable to respondents in the local language. The household survey collected information on the socio-demographic and economic characteristics of study respondents, health-seeking behavior around maternal and newborn care, access and use of maternal health voucher scheme, geographic location of households and respective health facilities, etc.

Four sub-districts where the MHVS is being implemented were chosen as study sites from two divisions of the country, which are low-performing in terms of some MNCH indicators when compared to national rates [6]. The percentage of women receiving any ANC was 78.4% at the national level whereas for Chattogram and Sylhet this rate was 74.4% and 62.8% respectively in 2014. The two sites were also lagging behind in terms of facility-based delivery which was 37.4% at the national level and for Chattogram and Sylhet the rates were 35.2% and 22.6% respectively [25].

**Fig 1:**
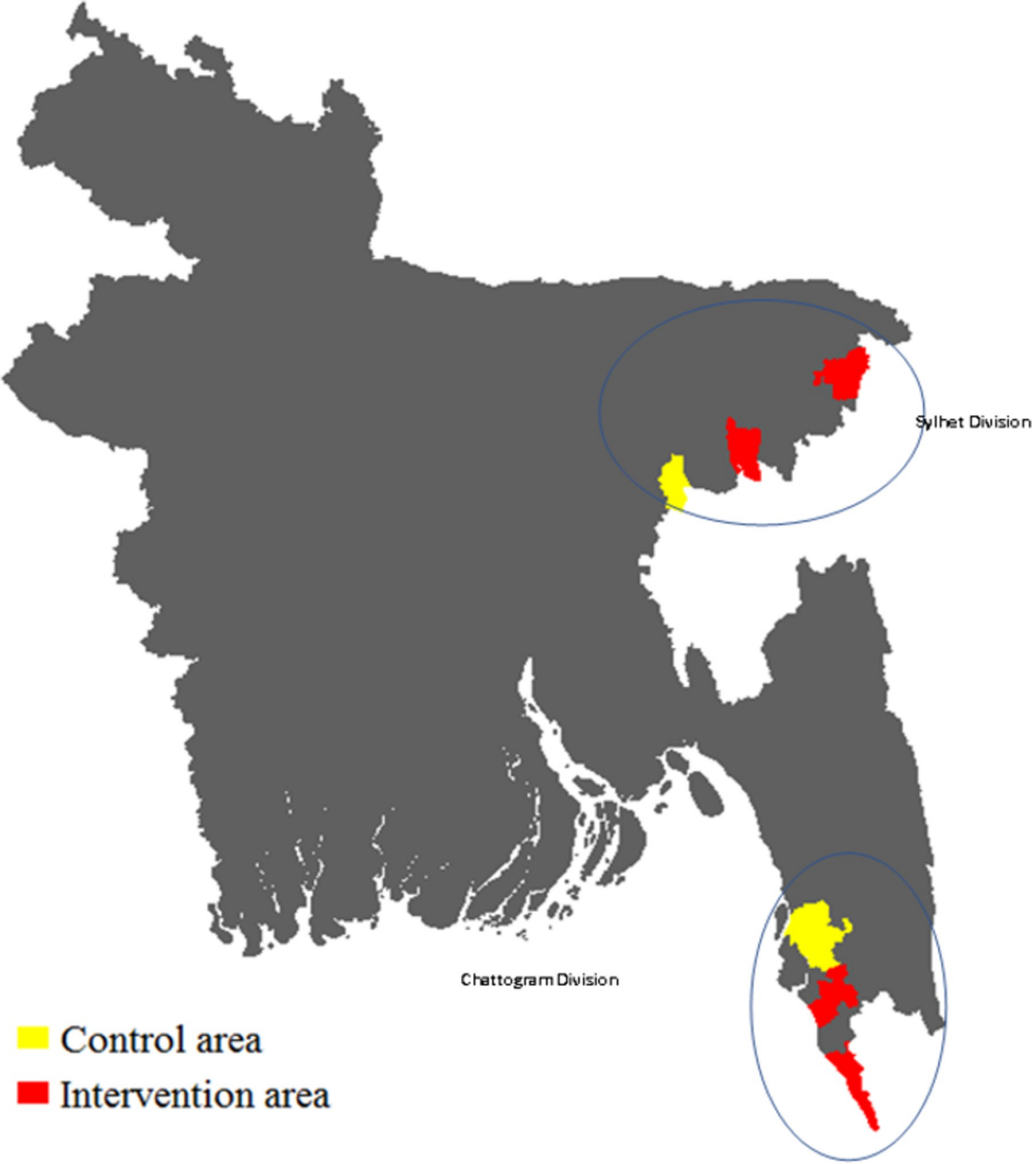
Study sites.

Ramu and Teknaf sub-districts were selected from the Chattogram division which is situated in the south-east region of the country and Sreemangal and Sulla sub-districts from the Sylhet division situated in the north-east region of the country (see Fig 1). Each of the selected sub-districts was divided into two parts–municipality (i.e. central area of the sub-district) and non-municipality areas. Municipality areas are within 5 km of Upazila Health Complexes (UHC) and/or other health facilities adjacent to it. Based on the study objective and earlier evidence, non-municipality areas were further broken down into two categories, villages within 5 km of a UHC and/or other health facilities adjacent to it and those located at a distance of more than 5 km away from a UHC and/or other health facilities adjacent to it. We used the 5 km cut-off following the World Health Organization (WHO) recommendation which states that for optimal access to healthcare everyone should live within a 5 km radius of a health facility [26]. The existing arrangement of services at the sub-district level shows that a majority of large-scale public, private, and NGO health facilities are located within 5 km of an Upazila Health Complex [11]. The health facilities of the study area were a mix of public, private, and NGO facilities. A detailed distribution of health facilities in the study areas is recorded in the facility registry maintained by the Director General of Health Services of the Ministry of Health and Family Welfare [27].

### Data

#### Household listing and sample survey

Considering 31% as the facility delivery rate in MHVS areas [16], to detect a difference of 10% in facility delivery with a 5% level of significance and 90% power, a sample size of 523 samples in each of the 4 MHVS intervention sub-districts were required. Accounting for non-response and attrition, it was planned to conduct face-to-face interviews of 600 women in each of the sub-districts coming to a total of 2400 women from four study sub-districts.

The data collection team consisted of one male and one female Field supervisor and twenty interviewers (Research Assistants). Field Supervisors were recruited from the head office and interviewers were recruited locally based on their previous interview conduction and supervisory experience considering their capacity to understand the local language quickly. Interactive training sessions were organized to discuss study objectives, the questionnaire, sampling data collection, and management techniques. After the training, field testing of the questionnaire along with pilot interviews were carried out. Interactive discussions were then held to discuss lessons learned, challenges faced from the pilot interview, and how to overcome them.

Household listing was carried out in villages selected from four study sub-districts using the Probability Proportional to Size (PPS) method [28]. A total of 9587 households were listed of which 2389 from Ramu, 2390 from Teknaf, 2398 from Sreemangal, and 2410 from Sulla were selected.

**Fig 2:**
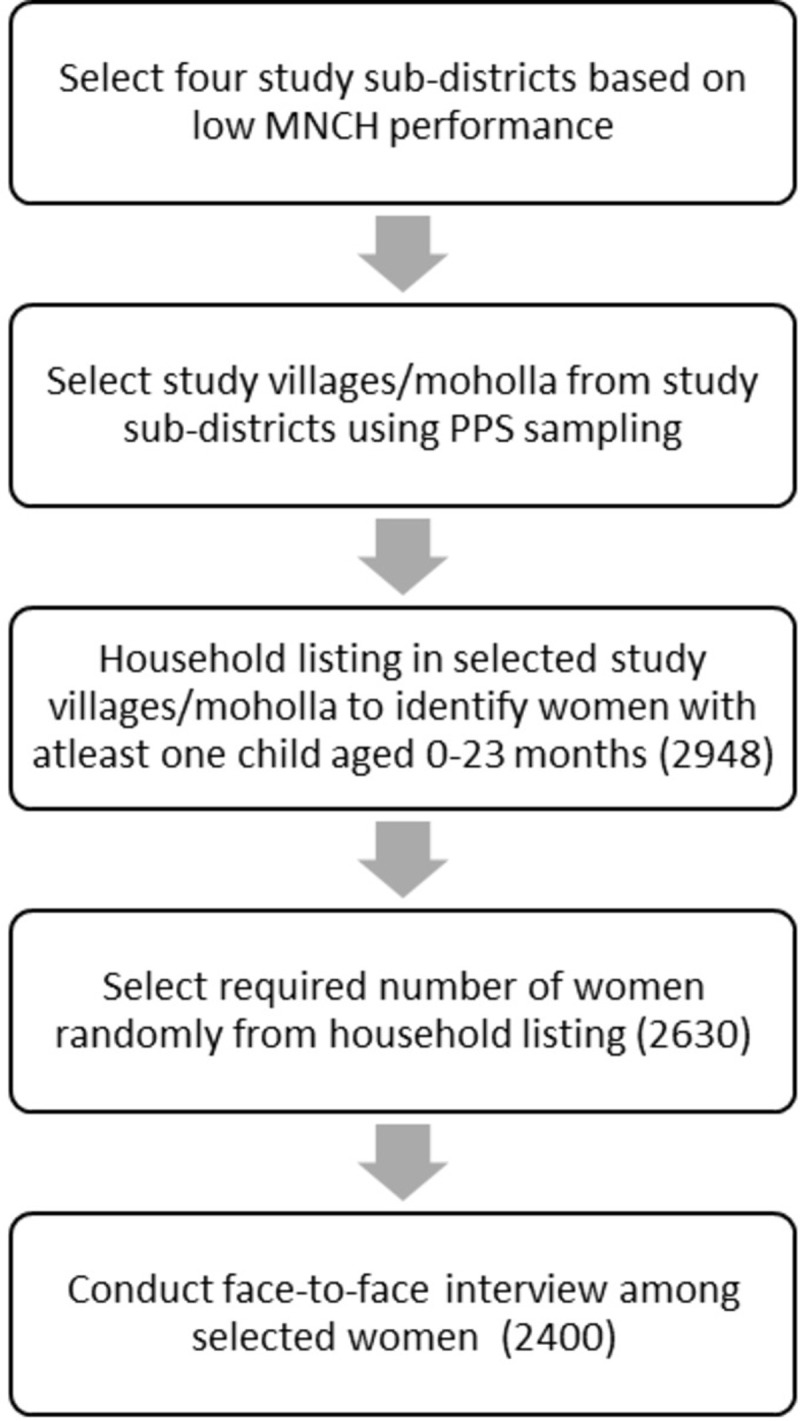
Flowchart of random selection of women.

Women who had at least one live birth within the past 24 months were considered eligible to become respondents for the study. From the household listing, 2948 eligible women were identified. For conducting face-to-face interviews, 2630 (out of 2948) randomly selected women were attempted. Finally, 2400 women (91% response rate (2400/2630)) were successfully interviewed (see Fig 2). Reasons for not participating in the survey were their reluctance to take part in the survey, and unavailability during the three-day attempts at selected mother’s house.

### Variables

The potential accessibility variable for coverage of maternal and newborn continuum of care in the study was taken as the distance from the mother’s residence to her local Upazila Health Complex.

#### Independent variables or covariates

Women who were members of the MHVS were termed ‘MHVS’ while those who were not members of the voucher scheme were termed ‘Non-MHVS’. For analytical purposes, the variable regarding distance to UHC from villages where women reside was recoded into two categories, within or further than 5 km from the UHC. Households from where women were chosen for interview were all GIS mapped. We also recorded the GIS code of all upazila health complexes from the study areas which allowed us to estimate the exact distance of women’s residences from the health facilities in kilometers. Categorization of the variables ‘birth order’ and ‘mother’s age at birth’ were done based on the median scores. Using data for asset with substantial frequency such as–ownership of household assets, source of drinking water, types and sharing of toilet facility, ownership of livestock, ownership of homestead and land, source of cooking fuel, the main material of floor, roof, and wall, the average number of people sleeping in one room, a wealth index as a proxy measure of economic status was created in tertiles (i.e. distributing one-third respondent in each of three categories) applying principal component analysis [21, 29, 30]. In the absence of income data, we used asset tertiles to identify women from poor households. Mother’s education was categorised as no education, primary (who passed class 1 to 5), secondary/higher (who passed class 6 or higher). Sub-district variable had four categories representing four study sites. The sex of children variable was categorized as male and female and was used as a covariate in the model assuming it may have an association with outcome variable utilization of Postnatal care (PNC) as part of MNCoC.

#### Dependent variables or outcome of interest

Dependent or outcome variables were categorized as binary variables for each stage of the maternal and newborn continuum of care (MNCoC). Utilization of antenatal services was categorized as those women who have received at least four versus fewer than four antenatal visits [31]. Facility delivery was considered a binary variable with delivery taking place at either public or private or NGO health facilities versus deliveries that occurred at home. A Skilled Birth Attendance variable was created by combining those women whose last live birth in the last 2 years was attended by any of the following providers–qualified doctor, nurse/paramedic/ midwife, Family Welfare Visitor (FWV), Community Skilled Birth Attendant (CSBA) versus those women whose birth was attended by none of above providers [6].

Variables regarding postnatal care received from a qualified provider within 2 days of delivery were created by grouping those women who received postnatal services for themselves or their children within 2 days of delivery from any qualified providers (e.g. qualified doctor, nurse/paramedic/midwife, FWV, CSBA, Medical Assistant (MA)/Sub-Assistant Community Medical Officer (SACMO)) versus those women who did not receive these services from any qualified provider [6].

It was considered that a mother was a recipient of the continuum of maternal and newborn care if she received all of the following services ‐ at least 4 antenatal visits, facility delivery or skilled birth attendance, and postnatal care from trained providers within 2 days of delivery [32].

### Analysis

Bivariate and multivariate analyses were carried out to the explore absolute and relative impact of independent variables on outcome variables.

In Bivariate analysis, we examined associations or absolute impact of major independent variables i.e. voucher membership status and distance to facility or upazila health complex (UHC) with outcome variables i.e. utilization of individual stage and continuum of maternal and newborn care (at least 4 antenatal visits, a skilled birth attendant, in-facility delivery, postnatal care from a qualified provider within 2 days of delivery). As a measure of association, we used the Pearsons chi-square test statistic [33]. CHISQ command is used in SPSS to get the calculated value of this test statistic along with the p value.

For multivariate analysis, to have a parsimonious model, service utilization is modeled using binary logistic regression following a stepwise method [34]. Significant predictor variables obtained from bivariate analysis and other related important factors based on related study evidence [21] were considered as potential independent variables in the multivariate analysis.

The Dependent or response variable was defined as binary contrasts, which take the value of 1 if a woman accessed the continuum of maternal and newborn care, and zero otherwise.


$${}_{\begin{array}{l} {\text{y}}_{\text{i}}=1,\;\text{if}\;\text{continuum}\;\text{of}\;\text{maternal}\;\text{and}\;\text{newborn}\;\text{care}\;\text{is}\;\text{used}\\ \;=0,\;\text{if}\;\text{continuum}\;\text{of}\;\text{maternal}\;\text{and}\;\text{newborn}\;\text{care}\;\text{not}\;\text{used} \end{array}}$$

We modeled the probability of utilizing continuum of maternal and newborn care which we defined as π_*i*_ = Pr(*y*_*i*_ = 1) in the form of the regression equation

$$\text{ln}(\frac{\pi_{\text{i}}}{1-\pi_{\text{i}}})=\beta_{0}\;+\;\beta_{1}{\text{Voucher}}_{\text{i}}\;+\;\beta_{2}{\text{Distance}}_{\text{i}}\;+\;\beta_{3}{\text{Voucher}}_{\text{i}}\;*\;{\text{Distance}}_{\text{i}}\;+\;{\gamma \text{z}}_{\text{i}}$$

where β_1_ and β_2_ represent the coefficient for the effect of voucher and effect of distance to UHC respectively on the probability of continuum of maternal and newborn care utilization, with the independent variables *Voucher*_*i*_ and *Distance*_*i*_ respectively being defined as 0/1 contrasts as to whether woman *i* received a voucher, and whether she lived within 5 kilometers of UHC. The effect of other characteristics (mother’s education, wealth index, sex of children, parity/birth order, and mother’s age at birth) are captured in the vector of control variables z_i_.

The multivariate model is specified as the main and interaction effect model, which captures the effect of voucher receipt and distance to UHC or health facility along with other potential confounders on service utilization. An interaction between voucher membership status and distance to UHC or health facility (Voucher_i_ *Distance_i_) was also considered to be included in the model to test whether the effect (coefficient β_3_) of the voucher membership on utilization of the continuum of maternal and newborn care varies between within 5 km and beyond 5 km of UHC compared to that of non-voucher mother. Other independent variables as control variables were included in two models (one with all potential confounders excluding socioeconomic status variables and another one with all potential confounders including socioeconomic status variables) to ensure these effects are robust to women’s and children’s socioeconomic and other background characteristics. Predictor variables were included or excluded from the model step by step as per the criteria of the stepwise model selection procedure such as probability in (i.e. p<0.05) and probability out (i.e. p>0.10). Likelihood ratio tests (LRT) were used to understand the goodness-of-fit of the models as well as to understand the significance of the odds ratio associated with each category among the independent variables.

## Results

### General description of study population

Table 1 depicts the background characteristics of the respondents. In total, there were 2400 respondents from four study sites (600 from each) who are women of at least 1 child aged 0–23 months. The median age of women was 26 years. 41% of the women had primary level education, 32% secondary level education and 27% of the women were illiterate.

**Table 1:** Background characteristics of respondents.

Variables	Voucher membership status		All
	MHVS	Non MHVS	
	% / Mean	% / Mean	% / Mean
**No. of women**	**458**	**1942**	**2400**
**Mother’s age (in years)**			
Mean ± S.D.	24.5 ± 4.7	27.0 ± 5.9	26.5 ± 5.7
Median	23.0	27.0	26.0
**Mother’s education**			
No education	17.7	29.0	26.8
Primary	38.2	41.9	41.2
Secondary/higher	44.1	29.1	32.0
**Wealth index**			
Low	22.1	36.0	33.3
Middle	39.5	31.9	33.3
High	38.4	32.1	33.3
**Mother’s age at birth (for last live birth in last 2 years)**			
≤25 years	69.0	49.0	52.8
>25 years	31.0	51.0	47.2
**Sex of children**			
Male	50.4	52.3	51.9
Female	49.6	47.7	48.1
**Birth order**			
≤2	82.8	54.0	59.5
>2	17.2	46.0	40.5
**Distance to UHC from villages**			
Within 5 km	30.1	21.0	22.8
Beyond 5 km	69.9	79.0	77.3

Note: S.D. = Standard deviation

53% of women delivered their last live birth in the last 2 years at the age of 25 years or below while 47% delivered after the age of 25 years. A slightly higher proportion of children was male (51.9%) compared to female (48.1%). Birth order of the last live birth in the last two years was two or below in the case of 59.5% of women. 19% of the women were members of the maternal health voucher scheme in four intervention study sites. 23% and 77% of respondents were from villages within and beyond 5 km of UHC respectively.

### Major findings of the study

For bivariate analysis we followed a procedure reported [21]. Based on bivariate analysis results Table 2 shows absolute comparisons in the use of maternal and newborn health services by voucher and non-voucher scheme women residing within and beyond 5 km of UHC. While exploring distance to health facility-related inequality within the voucher mother groups, no significant difference was observed in the utilization of 4 or more antenatal visits, skilled birth attendance, postnatal care from qualified providers within 2 days of delivery, continuum of maternal and newborn care between those who lived within 5 km of UHC and those residing beyond 5 km of UHC. Among voucher scheme women only the difference in utilization of facility delivery between those residing within and beyond 5 k.m. of UHC was found significant (p<0.001) which may be associated with flat rate routine transport cost benefit for visiting health facilities under the MHVS package. Among voucher scheme beneficiary women within to beyond ratio in the utilization of facility-based delivery care was found comparatively higher (1.4) than that of other maternal and newborn care.

**Table 2:** Utilization of maternal and newborn care by voucher membership status and distance to facility.

Indicators	MHVS				Non- MHVS			
	Within 5 km of UHC % (n/N) 95% C.I.	Beyond 5 km of UHC % (n/N) 95% C.I.	P value^ǂ^	Within to beyond ratio	Within 5 km of UHC % (n/N) 95% C.I.	Beyond 5 km of UHC % (n/N) 95% C.I.	P value^ǂ^	Within to beyond ratio
At least 4 ANC	62.3 (86/138) 54.3–70.3	56.9 (182/320) 51.4–62.1	0.278	1.1	40.4 (165/408) 35.8–45.3	31.5 (483/1534) 28.9–34.0	0.001	1.3
Facility delivery	63.8 (88/138) 55.9–71.5	45.6 (146/320) 40.4–51.5	<0.001	1.4	34.6 (141/408) 29.4–38.9	21.3 (326/1534) 19.2–23.2	<0.001	1.6
Skilled birth attendance	63.8 (88/138) 55.2–71.4	54.1 (173/320) 48.9–59.7	0.054	1.2	42.6 (174/408) 37.8–46.9	25.7 (394/1534) 23.5–27.9	<0.001	1.7
PNC from qualified providers within 2 days of delivery	62.3 (86/138) 53.8–70.2	55.9 (179/320) 50.7–61.5	0.204	1.1	40.9 (167/408) 36.0–45.7	28.6 (438/1534) 26.3–30.9	<0.001	1.4
Continuum of maternal and newborn Care	38.4 (53/138) 30.0–46.9	33.4 (107/320) 28.5–38.8	0.306	1.2	24.3 (99/408) 20.3–28.3	11.5 (176/1534) 9.9–13.1	<0.001	2.1

Note: ǂ = Based on Pearson’s chi-square for difference in utilization of maternal and newborn care between women residing within and beyond 5 km of UHC; UHC = Upazila Health Complex

While exploring distance to health facility-related inequality within the non-voucher mother group, significant (p<0.001) differences were observed in utilisation of 4 or more antenatal visits, facility delivery, skilled birth attendance, PNC from qualified providers within 2 days of delivery, continuum of maternal and newborn care between those who lived within 5 km of UHC and those residing beyond 5 km of UHC. Among non-voucher scheme women within to beyond ratio in utilization of maternal and newborn continuum of care was found comparatively higher (2.1) than that of other maternal and newborn care services.

From the bivariate analysis results differences in utilization of maternal and newborn care between women residing within and beyond 5 k.m. of UHC were found higher (especially much higher for utilization of MNCoC) among non-voucher women than that of voucher women with some exceptions regarding utilisation of facility delivery (Table 2). Hence, we have considered utilisation of MNCoC as an outcome variable in multivariate analysis which covers all three components of the continuum of maternal and newborn care.

### Findings of advanced analysis

In Table 3 results of stepwise logistic regression analysis from two fitted models with main and interaction effects including and excluding socio-economic status variables are presented. All main and one interaction effect variables from the two models came out to be significant in the analyses: voucher membership status, distance to UHC from villages, the interaction between MHVS membership status and distance to UHC, wealth index, mother’s education, mother’s age at birth, sex of children, birth order. Besides, prior to fitting the multivariate stepwise logistic regression model along with the outcome variable ‘utilization of continuum of maternal and newborn care’ and each of the concerned independent variables separate models were fitted from where unadjusted odds ratios, 95% confidence intervals, and level of significance (p values) were calculated to understand their absolute impact and these results are presented in the S1 Table.

**Table 3:** Odds ratios of distance to UHC and other factors associated with utilization of maternal and newborn continuum of care (Main and interaction effect model).

Independent variables	Adjusted odds ratios (95% CIs) (Excluding wealth index)	P value	Adjusted odds ratios (95% CIs) (Including wealth index)	P value
Voucher membership status***		0.000		0.000
Non-MHVS (r)	1		1.0	
MHVS	3.130 (2.325–4.214)	0.000	3.165 (2.329–4.300)	0.000
Distance to UHC***^,^**		0.000		0.001
Beyond 5 km (r)	1		1.0	
Within 5 km	2.234 (1.673–2.983)	0.000	1.658 (1.225–2.243)	0.001
Wealth index***	-	-		0.000
High (r)			1.0	
Low			0.175 (0.120–0.254)	0.000
Middle			0.446 (0.341–0.583)	0.000
Mother’s education***		0.000		0.000
Secondary/Higher (r)	1.0		1.0	
No education	0.217 (0.155–0.304)	0.000	0.387 (0.271–0.554)	0.000
Primary education	0.360 (0.281–0.462)	0.000	0.507 (0.391–0.658)	0.000
Mother’s age at birth**		0.001		0.001
≤ 25 years (r)	1.0		1.0	
> 25 years	1.624 (1.233–2.138)	0.001	1.600 (1.207–2.121)	0.001
Sex of children*		0.016		0.013
Male (r)	1.0		1.0	
Female	0.759 (0.607–0.950)	0.016	0.747 (0.593–0.940)	0.013
Birth order***		0.000		0.000
≤ 2 (r)			1.0	
> 2	0.493 (0.363–0.668)	0.000	0.497 (0.365–0.678)	0.000
Voucher membership status by distance to UHC*		0.019		0.044
Women who are member of MHVS and reside within 5 km of UHC	0.537 (0.318–0.904)	0.019	0.577 (0.338–0.987)	0.044
Likelihood ratio test (LRT) statistic (-2loglikelihood)	1978.057		1873.251	

Note: r = Reference category; Significant at

*p<0.05

**p<0.01

***p<0.001; UHC = Upazila Health Complex

The existence of a difference in access to the continuum of maternal and newborn care between women residing within and beyond 5 km of UHC was significantly 0.6 times less likely for voucher women than of non-voucher women. MHVS recipients and those residing within 5 km of their local UHC were respectively 3.2 and 1.7 times more likely to access the continuum than their counterparts. Women from low and middle wealth index categories had respectively 0.2 and 0.5 times lower odds of MNCoC utilization compared to women from higher wealth index categories. Likewise, women with no education and those with primary level education had 0.4 and 0.5 times lower odds respectively compared to those with secondary or higher levels of education. Further, the use of MNCoC was less likely among women with a girl child (AOD 0.8) and of birth order more than 2 (0.5) compared to their counterparts. Women with age at first birth being more than 25 had a 1.6 times higher odds of MNCoC utilization compared to their counterparts (Table 3).

From the model with potential confounders excluding socioeconomic status variable, it was revealed that the difference in utilization of continuum of maternal and newborn care between mothers of MHVS residing within and beyond 5 k.m. of UHC was found to be significantly 0.5 times less likely compared to the mothers not having MHVS membership. Likelihood ratio test (LRT) indicate inclusion of socioeconomic status variable influence the model significantly.

## Discussion

The current study analyzed factors associated with the utilization of different components of maternal and neonatal care and more specifically with the Maternal and Neonatal Continuum of Care. The bivariate analysis showed that the use of maternal and neonatal care services and the MNCoC was similar for all voucher members irrespective of the distance between their residence and the health facilities except for facility delivery. On the other hand, for non-voucher members significant differences were observed for all components of maternal and neonatal care and the MNCoC between those living further away from health facilities and those living closer. The rates were much lower for those living further away indicating a significant distance-related inequality among non-voucher women.

However, it should also be mentioned that despite the positive influence on the reduction of distance-related inequality, the rate of service use among voucher members was lower than optimal; MNCoC for voucher members reaching only 38% and individual components of maternal and neonatal care ranging between 45.6% to 63.8%. The reasons behind this low rate of service use, particularly for MNCoC, need to be further explored to ensure the benefits of the voucher scheme reach its target population and the goal of universal coverage of maternal and neonatal care is achieved.

In the face of the current almost stagnant rate of maternal mortality in Bangladesh for the last two decades, MNCoC has been considered to be an important determinant of safe motherhood practices. Earlier studies have indicated that women using the complete CoC were more likely to experience positive maternal outcomes (e.g. facility-based delivery, skilled birth attendance, PNC use, and so on) [14].

Given the importance of MNCoC, the current study explored the determining factors of MNCoC use among voucher and non-voucher women. The findings from the multivariate analysis suggest that along with voucher membership, distance to health facilities from villages, mothers’ socioeconomic status, education, age at first birth, sex of children, and birth order, all had significant associations with utilization of MNCoC. The analysis explored the association of interaction term between distance and membership of MHVS which turned out to be significant and negative. This may indicate a narrowed distance-related inequality in MNCoC use among voucher members.

The positive relation of MHVS with reduced distance-related inequality regarding access to and utilization of a continuum of maternal and newborn care is notable: this association is more substantial than that on individual components of maternal and newborn care services. The influence is retained after controlling for other variables that are known to be determining factors of healthcare access such as educational attainment and socioeconomic status.

Evidence from earlier studies indicates that distance to a health facility and transport availability and cost are significant barriers to access to facility-based care [9, 11, 35]. Studies conducted on conditional cash transfer and/or voucher programme approaches tied to service use showed a positive impact of these schemes on the use of antenatal care, skilled birth attendance, and postnatal care [32]. Whilst voucher schemes potentially provide some means of relief for women in terms of the costs associated with accessing care, there are concerns over the effectiveness of the voucher schemes. Keya and colleagues found that the transport incentive element of a voucher scheme was not sufficient to cover costs in many cases where patients had to spend on average around four times more than the incentive value [22]. This is more of a concern for women living in remote regions. Distance thus plays a significant role in terms of accessing all components of services to achieve the continuum of maternal and newborn care which inevitably requires repeated visits to health facilities [9].

The MHVS provides a flat payment of BDT 100 ($1.4) to cover the transportation cost for each visit with a ceiling of four visits. The present findings indicate that as a priority, the MHVS scheme administrators should revise the transport incentive to accommodate higher reimbursement for remote areas (based on road conditions, availability, and cost of local transport) in order to enhance the impact of MHVS on service use and attainment of the continuum of maternal and neonatal care. We recommend consideration of alternative mechanisms for reimbursement of transport fees to reduce the geographical access barrier such as reimbursement based on distance or community-based transport models with revolving funds to ensure that maximum returns are achieved from national investment in the voucher scheme.

According to the Tanahashi framework five successive sequential stages of service coverage, namely availability, accessibility, acceptability, contact, and effectiveness, need to be equally addressed while designing any health interventions to improve access to healthcare [36]. Following this framework factors like availability of care and quality of care need to be explored in the future to further elaborate on the effect of MHVS on the use of maternal care and maximize its effectiveness.

While exploring the socioeconomic characteristics of the MHVS members we noticed the presence of a significant number of beneficiaries from higher socioeconomic status whereas the MHVS was meant for the low-income population. This points to the challenge of mistargeting of the scheme which limits the effectiveness of the scheme in enhancing the use of maternal and neonatal care services among this particular group of population.

In addition to the challenge of mistargeting, the current study also identifies gaps in maternal and neonatal healthcare service use which warrants further in-depth exploration to modify or redesign the current voucher scheme in order to maximize its reach and efficiency. In this relation, qualitative research could be designed to understand maternal and neonatal care-seeking behavior, particularly among low-income rural populations, the reasons for low utilization of maternal and neonatal care, and the experience of beneficiary and non-beneficiary women in accessing maternal and neonatal care which can eventually feed into modification or redesign of the current scheme.

## Conclusion

The findings from the current paper emphasize the potential positive influence of MHVS on maternal and neonatal care utilization. The current study findings support the scaling up of the scheme in the remaining sub-districts of Bangladesh to facilitate improved access to and utilization of maternal and newborn continuum of care. Demand-side financing mechanisms like the MHVS have the potential to support the achievement of universal coverage of maternal and newborn care across the continuum of care, particularly in regions where geographic or distance-related inequality is a major challenge.

### Strengths and limitations of the study

The strength of the study lies in its concentrated analysis of distance-related inequality in access to maternal and newborn care. Literature review indicates there are several articles regarding impact evaluation, and lessons learned from the voucher scheme but there is very little scientific evidence regarding the role of the voucher scheme in reducing distance inequality in maternal and newborn health service utilization. Another strength of the manuscript is that it provides very recent evidence on the role of voucher schemes in reducing distance inequality regarding the utilization of a continuum of maternal and newborn care. The limitation of the study lies in the fact that the data collection was carried out in the low-performing divisions of the country and hence the findings are more applicable to the settings where utilization of maternal and newborn care is comparatively lower. Having said that the significance of the findings is greater for this reason as the impact is expected to be greater for areas where the maternal and newborn health indicators are adverse. The study used the wealth index as a proxy measure of socioeconomic status created from the asset ownership data of respondents instead of income data as income data are not very reliable in settings such as this.

One other limitation lies in the fact that the use of MNCoC and all individual component of maternal and neonatal care was high among MHVS members compared to non-MHVS members in all settings which could be a result of the influence of other incentives offered in the scheme (e.g. free consultation, free normal and C-section delivery with facility delivery incentive). The higher socioeconomic status of a segment of the voucher recipients compared to the non-voucher women might have some influence also. However, due to the focus of the study towards distance-related inequality, these factors were not explored in detail. Lastly, the data was cross-sectional in nature which limits drawing specific causal relationships.

## Supporting information

S1 Table Unadjusted Odds ratios of distance to UHC and other factors associated with utilization of maternal and newborn continuum of care (Main and interaction effect model). (DOC)

S1 File (DOCX)

## List of abbreviations

MHVS: Maternal Health Voucher Scheme
UHC: Upazila Health Complex
MNCH: Maternal, newborn and child health
OOP: Out-of-pocket expenditure
ANC: Antenatal care
PNC: Postnatal care
Km: Kilometer
