# Supplementary material for: Does maternal health voucher scheme have association with distance inequality in maternal and newborn care utilization? Evidence from rural Bangladesh
Source: PLoS One. 2023 Dec 7;18(12):e0295306. doi: 10.1371/journal.pone.0295306 (PMC10703241; doi:10.1371/journal.pone.0295306)
Supplement: S1 Table — (DOC) [file pone.0295306.s001.doc]

**S1 Table: Unadjusted Odds ratios of distance to UHC and other factors associated with utilization of maternal and newborn continuum of care (Main and interaction effect model)**

| **Independent variables** | **Unadjusted odds ratios (95% CIs)** | **P value** |
| --- | --- | --- |
| Voucher membership status***  Non-MHVS ( r )  MHVS | 1.0  3.255 (2.584-4.099) | 0.000  0.000 |
| Distance to UHC***  Beyond 5 km ( r )  Within 5 km | 1.0  2.142 (1.709-2.684) | 0.000  0.000 |
| Wealth index***  High ( r )  Low  Middle | 1  0.106 (0.075-0.150)  0.360 (0.283-0.458) | 0.000  0.000  0.000 |
| Mother’s education***  Secondary/Higher ( r )  No education  Primary education | 1.0  0.174 (0.126-0.240)  0.306 (0.241-0.388) | 0.000  0.000  0.000 |
| Mother’s age at birth*  ≤ 25 years ( r )  > 25 years | 1  0.777 (0.630-0.959) | 0.019  0.019 |
| Sex of children*  Male ( r )  Female | 1  0.796 (0.646-0.981) | 0.033  0.033 |
| Birth order***  ≤ 2 ( r )  > 2 | 1  0.433 (0.343-0.547) | 0.000  0.000 |
| Voucher membership status by distance to UHC**  Women who are member of MHVS and reside within 5 km of UHC | 0.502 (0.305-0.826) | 0.007  0.007 |
| Note: r = Reference category; Significant at *p<0.05, **p<0.01, ***p<0.001; UHC = Upazila Health Complex | | |
