# Supplementary material for: Does maternal health voucher scheme have association with distance inequality in maternal and newborn care utilization? Evidence from rural Bangladesh
Source: PLoS One. 2023 Dec 7;18(12):e0295306. doi: 10.1371/journal.pone.0295306 (PMC10703241; doi:10.1371/journal.pone.0295306)
Supplement: S1 File — (DOCX) [file pone.0295306.s002.docx]

Date: December, 2022

To

PLOS ONE

**Subject: Request for PLOS Publication Fee Assistance for full waiver of the article-processing charge**

Dear Team,

I am pleased to inform you that Dr. Shehrin Shaila Mahmood, Associate Scientist, Health Systems and Population Studies Division at icddr,b is processing submission of a manuscript titled “Does the maternal health voucher scheme have association with distance inequality in maternal and newborn care utilization? Evidence from rural Bangladesh” as the corresponding and joint first author. The paper is based on findings from a project funded by MRC-UK and the project was completed in June 2021.

After reviewing the budget of the project, I confirm that, Dr. Mahmood does not have access to any funds from this project to support payment of publication charges. Further, icddr,b being an institute based in an LMIC, she also does not have access to any funding from icddr,b for this purpose.

Therefore, we would be very grateful if you allow full waiver of the article-processing fee for this manuscript.

Sincerely yours,


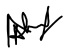


Armana Ahmed

Head, Research Administration
